# All inequality is not equal: children correct inequalities using resource value

**DOI:** 10.3389/fpsyg.2013.00393

**Published:** 2013-07-19

**Authors:** Alex Shaw, Kristina R. Olson

**Affiliations:** Social Cognitive Development Lab, Department of Psychology, Yale UniversityNew Haven, CT, USA

**Keywords:** fairness, value, inequity aversion, social norms, social exchange

## Abstract

Fairness concerns guide children's judgments about how to share resources with others. However, it is unclear from past research if children take extant inequalities or the value of resources involved in an inequality into account when sharing with others; these questions are the focus of the current studies. In all experiments, children saw an inequality between two recipients—one had two more resources than another. What varied between conditions was the value of the resources that the child could subsequently distribute. When the resources were equal in value to those involved in the original inequality, children corrected the previous inequality by giving two resources to the child with fewer resources (Experiment 1). However, as the value of the resources increased relative to those initially shared by the experimenter, children were more likely to distribute the two high value resources equally between the two recipients, presumably to minimize the overall inequality in value (Experiments 1 and 2). We found that children specifically use value, not just size, when trying to equalize outcomes (Experiment 3) and further found that children focus on the relative rather than absolute value of the resources they share—when the experimenter had unequally distributed the same high value resource that the child would later share, children corrected the previous inequality by giving two high value resources to the person who had received fewer high value resources. These results illustrate that children attempt to correct past inequalities and try to maintain equality not just in the count of resources but also by using the value of resources.

Fairness is certainly important to human society (Boyd and Richerson, 2005), but deciding how best to be fair is no easy task. Adults and children must balance many different obligations and norms when deciding what is fair: they need to consider past inequalities, reciprocity, the value of resources, social relationships, and the amount of work that others have done when deciding how to fairly distribute resources (Fiske, 1992; Mills and Clark, 1994; Fehr and Schmidt, 1999; Olson and Spelke, 2008; Moore, 2009; Shaw and Knobe, 2013). Indeed, sometimes doing the fair thing requires, counter intuitively, distributing resources unequally. For example, imagine two employees both did a good job on a project and their boss rewarded them with baseball tickets, but one employee was given three tickets while the other was given only one. It would be fair for the boss to give two additional baseball tickets to the employee who had received fewer tickets, at a later date, but it would not be fair for the boss to give the employee two new company cars to address the ticket-based inequality. Giving unequally is fair in the former case because it corrects the past unequal distribution, but not in the latter case because this would over-correct the past inequality, actually increasing the overall inequality. The reason for this difference is that a car is substantially more valuable than a ticket to a baseball game. Adults recognize that all inequalities are not equal; they will share unequally themselves in order to correct or minimize inequality between others (Dawes et al., 2007; Xiao and Bicchieri, 2010), and do so by taking resource value into account (Cook and Hegtvedt, 1983; Brown, 1984; Gurven, 2006).

One goal of the current research is to examine whether children focus on the norm of sharing equally themselves, or on trying to make the overall distribution of resources equal by correcting existent inequalities. If they do correct previous inequalities in order to equate outcomes, a second goal of the current research is to investigate whether children take resource value into account when trying to minimize inequality between others. Do they correct inequalities by trying to make the count of resources equal—giving cars to make up for unfair ticket-giving—or do they attempt to make the overall value of the resources as equal as possible?

We know that children are biased toward equal distribution of resources, but there has been very little research on how children respond to existent inequalities. Research with infants using looking time measures suggests that by the second year of life infants expect resources to be distributed equally between two agents, as long as both agents are highlighted as possible recipients (Geraci and Surian, 2011; Schmidt and Sommerville, 2011; Sloane et al., 2012; Sommerville et al., 2012). By 3 to 4 years of age, children themselves share resources equally with third parties when they can (Olson and Spelke, 2008), and are reluctant to share unequally when an equal option is possible, even when they know that one recipient was mean in a previous interaction (Kenward and Dahl, 2011), or did more work than the other recipient (Baumard et al., 2012). Indeed, if an equal option is possible, children default to giving equally rather than based on merit until they are 6 years old (e.g., Lerner, 1974; Hook and Cook, 1979; Sigelman and Waitzman, 1991). We also know that by age six children dislike those who share unequally (Shaw et al., 2012). While we know that by age six children will distribute resources unequally themselves based on merit and dislike those who share unequally, we do not know if children will distribute resources unequally themselves to correct previous inequalities in service of making overall outcomes equal [the one exception is Libby and Garrett (1974), but this experiment conflates correcting previous inequalities with reciprocity]. Because in the real world not everyone will always share equally, it is important to know how children respond to existent inequalities. Do they simply maintain these inequalities by trying to share equally themselves, or do they attempt to correct these inequalities by sharing unequally? This is one question the current research will address.

We also know very little about how children divide resources that differ in value, despite the fact that many forms of exchange involve resources that are not equal in value. Most research on children's and even adults' equality concerns has focused on decisions that involve distributing a single type of resource, for example, distributing a sum of money or a sum of cookies rather than having a person divide some money and some cookies (for reviews, see Damon, 1977; Walster et al., 1978; Hook and Cook, 1979). Using a single resource is useful because it minimizes random variation in preference for different resources. However, using a single resource fails to capture an important aspect of real world exchanges, and certainly does not capture exchanges in pre-agrarian human societies since fungible currency is a relatively recent human invention (Burgoyne and Lea, 2006). Indeed, there are very few fungible resources—one cannot substitute a unit of iPod for a unit of yoyo. Equally sharing non-fungible resources requires some recognition of value: how many units of resource A could be exchanged for how many units of resource B (Fiske, 1992). Being able to equate the value of varied resources is not only important in modern societies, but is also important for bartering and food sharing in smaller hunter-gatherer societies—even within the same animal carcass, different regions of the animal vary in value (Hill and Kaplan, 1993; Gurven, 2004). Since real world exchanges require some understanding of value, it is important to understand how value influences children's intuitions about how to share with others.

What little work that has been done on children's understanding of value has examined how children's preference for a resource influences their willingness to give resources to another person. We know that children, like adults, demonstrate preferences for some goods over others (Harbaugh et al., 2001), and use their preferences when deciding how many resources to give away (Birch and Billman, 1986). Blake and Rand (2010) had 3- to 6-year-old children identify their least favorite sticker (Low Value) and their most favorite sticker (High Value). They found that children shared more of their least favorite sticker than their most favorite sticker [for a similar effect in adults, see Novakova and Flegr (2013)]. This result importantly demonstrates that children weigh the value of being generous or fair against the personal value they place on a resource. However, this result does not tell us if children take value into account when deciding how to minimize inequality between others. Doing so requires children to use their preferences or some other information to make guesses about what others would want, and try to minimize inequality between others based on this dimension. This assumes that children have a belief about the value of resources that goes beyond their own idiosyncratic preferences. If children really understand value, they should distribute resources to two third parties in a way that minimizes the discrepancy in the value of the resources that the two third parties have. In terms of the example above, if children believe that it is inappropriate to give someone two company cars to correct a past inequality of two baseball tickets, this would suggest that they understand value and use it to guide their judgments.

## Experiment 1

In Experiment 1, we first investigated whether children correct existent inequalities in order to minimize inequality in outcomes. Children were asked to share two resources with two non-present recipients who had already received resources from the experimenter. The experimenter gave three resources to one of the recipients and one to the other recipient. If children try to make outcomes equal, then they should give both erasers to the recipient with fewer resources (giving unequally but correcting the inequality) rather than giving one to each recipient (maintaining the inequality by giving equally themselves). We investigated this question in 6- to 8-year-old children because past research has demonstrated that it is at this age that children become comfortable sharing unequally with third parties, at least based on merit (e.g., Hook and Cook, 1979; Shaw and Olson, 2012).

If children do give more resources to those who currently have fewer resources, it would be unclear if they do so in order to keep the count of the resources equal, or in order to keep the value of the resources equal. To investigate if children use value to determine how to equalize outcomes, we included two conditions in which children were sharing resources that were slightly more valuable (jar of Play-Doh, Medium Value Condition) or much more valuable ($20 bill, High Value Condition) than the resources that were initially shared unequally by the experimenter. If children want to keep the count of resources equal, then children should respond similarly in all conditions, by giving two resources to the recipient with fewer resources and thus equalizing the count of resources. If instead children care about keeping the value of resources as equal as possible, then, as the value of the resources to be shared increases, children should become increasingly likely to share equally themselves by giving one resource to each recipient. We investigated these questions in this study.

### Methods

#### Participants

Participants included 84 children aged 6 to 8 years old. Of these participants, 28 were in the Equal Value Condition (*M* = 7 years, 6 months, *SD* = 12 months; 15 females), 28 were in the Medium Value Condition (*M* = 7 years, 4 months, *SD* = 11 months; 8 females), and 28 were in the High Value Condition (*M* = 7 years, 0 month, *SD* = 12 months; 15 females).

#### Procedure

Two buckets were placed in front of the participant and the experimenter said (modeled on Shaw and Olson's (2012) method):

Thanks for playing this game with me. Earlier today, two kids named Mark and Dan did a great job cleaning up their room and we want to give them erasers as a prize. The problem is I don't know how much to give them; can you help me with that?

We are going to decide how many erasers Mark and Dan will get. Mark's erasers go in this bucket and Dan's erasers go in this bucket. We have six erasers. I am going to give these four erasers and you are going to give these two erasers. I'll go first. We have one for Mark, one for Dan. One for Mark, and one more for Mark. Now it's your turn; here are two erasers. Give them however you want.

Each time Mark or Dan's name was used, the experimenter pointed to the corresponding bucket. During the allocation phase of the task, the experimenter placed an eraser into the corresponding bucket when noting who was receiving the eraser (Mark or Dan). The erasers were colorful and shaped like fun things children like, such as turtles, sports balls, and ice cream cones, and have been used in previous research on decision-making in children (Shaw and Olson, 2012; Shaw et al., in press). After the allocation phase, children were handed two erasers that they could place in the two buckets however they wanted. Children were always given two resources to distribute, and distributed until there were no resources remaining. On half of the trials Mark's bucket was on the left, and on half of the trials Mark's bucket was on the right.

In order to investigate the influence of value on children's decisions, we had two additional conditions in which children distributed resources that were slightly more valuable (Medium Value Condition) or much more valuable (High Value Condition) than the resources (erasers) that were shared unequally by the experimenter; all other aspects of the design of these conditions was identical to the condition described above. The slightly more valuable object in the Medium Value Condition was a 3 oz jar of Play-Doh, and the much more valuable resource in the High Value Condition was a $20 bill. Children could not make the value of resources equal in these conditions, since both Play-Doh and a $20 bill are presumably worth much more than two erasers (for empirical verification that children see these items as more valuable than erasers, see Experiment 4), but they could ensure that the inequality did not increase. Specifically, they could give one high value resource to each recipient rather than giving two to the person with fewer low value resources, if they were interested in maintaining the smallest overall inequality.

### Results

Because no children chose the option of giving two erasers to the person with more erasers, we conducted analyses with just the two strategies that children used—sharing equally by giving one to each recipient, or giving two to the person with fewer resources. We first conducted a 3 × 2 Yates-corrected χ^2^ test on children's responses in the Equal, Medium, and High Value Conditions, which revealed a main effect of condition, χ^2^ (2, *N* = 84) = 23.37, *p* < 0.001.

**Figure 1:**
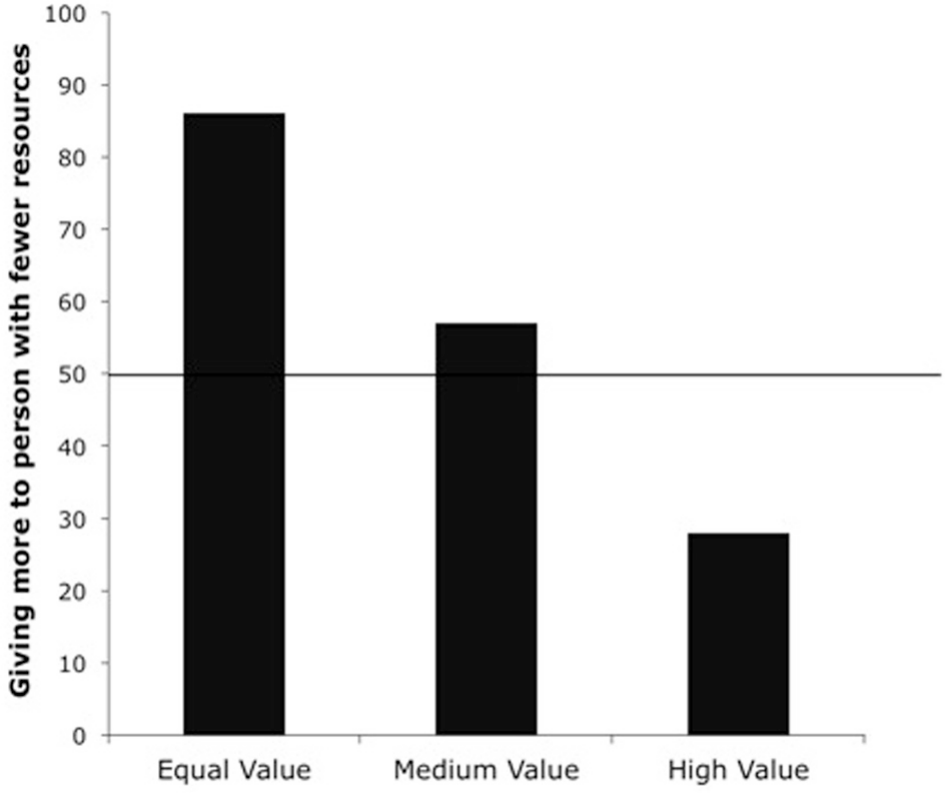
**Percentage of children giving two resources to the recipient with fewer resources in the Equal, Medium and High Value Conditions from Experiment 1**.

We then examined whether children's responses differed between pairs of conditions by conducting Yates-corrected χ^2^ tests. A 2 × 2 Yates-corrected χ^2^ test revealed that children in the Equal Value Condition were more likely to give two erasers to the disadvantaged recipient than children in the High Value Condition, χ^2^ (1, *N* = 56) = 23.17, *p* < 0.001, and children in the Medium Value Condition, χ^2^ (1, *N* = 56) = 4.29, *p* = 0.038 (see Figure 1). As the resources became more valuable than those involved in the original inequality, children were more likely to give one to each recipient than to give two to the recipient who had fewer resources. Next, we examined if children's responses differed in the Medium and High Value Conditions using a Yates-corrected χ^2^ test, which revealed that children in High Value Condition were more likely to give one resource to each child than children in the Medium Value condition, χ^2^ (1, *N* = 56) = 7.62, *p* = 0.006. Again, as the value of the resources increased, children shifted their preference from giving two to the recipient with fewer resources to giving one resource to each recipient.

We next conducted binomial tests to compare children's responses to chance responding. The binomial test on the Equal Value Condition revealed that children gave two to the recipient with fewer erasers (24 out of 28) more often than giving one to each recipient (4 out of 28), *p* < 0.001. This result indicates that children preferred to make the total amount of resources equal between recipients, rather than to give equally themselves, when all resources were of equal value. A binomial test on children's choices in the Medium Value condition revealed that children did not show a preference for how to distribute the medium value resources, with about half the children giving two to the recipient with fewer resources (16 out of 28), and about half of the children giving one to each recipient (12 out of 28), *p* = 0.572. However, the binomial test on the High Value Condition revealed that children gave one to each recipient (23 out of 28) more often than giving two to the recipient with fewer erasers (5 out of 28), *p* = 0.001.

### Discussion

Children corrected inequalities created by an experimenter, and did so by attempting to equate the value, not just the count, of the resources distributed. When children distributed resources that were equal in value to those shared unequally by the experimenter, children gave more resources to a recipient who had received fewer resources in order to correct the existent inequality. Children could have ignored the inequality that was created by the experimenter and simply focused on the norm of giving equally themselves, since we know from past research that children have a tendency to share equally with others (Damon, 1977). However, this result indicates that children can inhibit their tendency to give equally to others when they are confronted with someone who had received fewer resources previously. Rather than simply defaulting to giving one resource to each recipient, children wanted to ensure that both recipients received an equal number of resources, at least when those resources were of equal value.

We next asked whether, when children try to make outcomes equal, they try to simply make the count of resources equal, or whether they consider the value of the resources. Our results suggest that children do use value when deciding how to share. Children behaved differently when sharing resources that were much more valuable (e.g., $20 bills) than the unequally shared erasers, giving the resources equally themselves rather than attempting to correct the past inequality. Perhaps children distributed the more valuable resources differently because they realized that giving the disadvantaged child two $20 bills would actually make the outcome even more unequal, though now in the other recipient's favor.

Although we interpret these results as indicating that children minimize inequality between others by using value, this is not the only possible interpretation. One alternative possibility is that children were confused in the Medium and High Value Conditions because they were required to match distributions involving multiple resources. However, the fact that children differentiated between the Medium and High Value Conditions speaks against this alternative—the resources used in both the Medium and High Value Conditions were different from the resources that were distributed by the experimenter, yet children treated these two conditions differently, suggesting they used some sense of value to guide their decisions. However, this by itself does not provide enough evidence to rule out the possibility that children were confused in these conditions. Perhaps children were indeed confused in the Medium Value Condition, and only behaved differently in the High Value Condition because there is something special about money that makes children more likely to share equally or pay attention to the value of resources. Previous research with adults indicates that when people distribute money, as compared to other resources, they are likely to think in terms of market exchanges (DeVoe and Iyengar, 2010) and this may cause them to think about resources in terms of their value (Fiske, 1992). In Experiment 2, we controlled for this possibility by having children divide high value resources that were not money. We also attempted to further rule out the possibility that children responded differently in the higher value conditions because they were confused by having to distribute resources that were different from those shared by the experimenter. To do this, in Experiment 2, both conditions had children dividing resources that were different from the resources shared by the experimenter. However, in one condition children were sharing a higher value resource and in the other condition they were sharing a lower value resource. If children were merely confused in Experiment 1 by having to divide resources that were different than those shared by the experimenter, then they should respond similarly in both conditions of Experiment 2 because children are dividing different resources in both conditions. If instead, as we predict, children were using value to guide their decision of how to equate outcomes, then they should be less inclined to give one to each recipient when they are dividing a resource of lower value as compared to one of higher value.

## Experiment 2

### Methods

#### Participants

Participants included 56 children aged 6 to 8 years old. Of these participants, 28 were in the Lower Value Condition (*M* = 7 years, 5 months, *SD* = 10 months; 13 females) and 28 were in the Higher Value Condition (*M* = 7 years, 1 month, *SD* = 8 months; 19 females).

#### Procedure

The procedure for Experiment 2 was very similar to that used in Experiment 1. Again the experimenter gave out four resources unequally, giving three to one recipient and one to the other, using the script described in Experiment 1. Then, the participant was told to share two resources with the two recipients. We did, however, make two changes. First, we used different resources in Experiment 2. In the Higher Value Condition, the experimenter gave out four lower value resources (four small fruit-flavored candies) and the participant gave out two higher value rewards (two full-sized chocolate candy bars). This method was similar to the Medium and High Value Conditions from Experiment 1, so we predicted a similar pattern of results—that participants would be less willing to give more resources to the person who had fewer resources. In the Lower Value Condition, the experimenter gave out high value resources (four full-sized chocolate candy bars), and the participant gave out two lower value rewards (two small fruit-flavored candies). If children's responses in the previous experiment were merely being driven by confusion about how to distribute a resource different than the one involved in the original inequality, or by money priming them to think about value, then they should respond at chance or give one lower value resource to each recipient as they did in the Medium and High Value Conditions from Experiment 1. However, if children try to equate value to minimize inequality of outcomes between others, then they should instead give two low value resources to the recipient who received fewer higher value resources. Giving more low value resources to the recipient with fewer resources would not make the distribution equal, but is the most equal option available to children. We deliberately used different types of candy because we wanted to ensure that children were responding to value, not merely thinking about the resources in terms of large and small quantities of the same resource (for empirical verification that children see the chocolate bars as more valuable than the small fruit candies, see Experiment 4).

A second change from Experiment 1 to Experiment 2 was that we now presented the resources on pieces of paper (5 × 8″) rather than placing them in buckets. We modified this aspect of the design to reduce the memory load required to complete this task.

### Results

**Figure 2:**
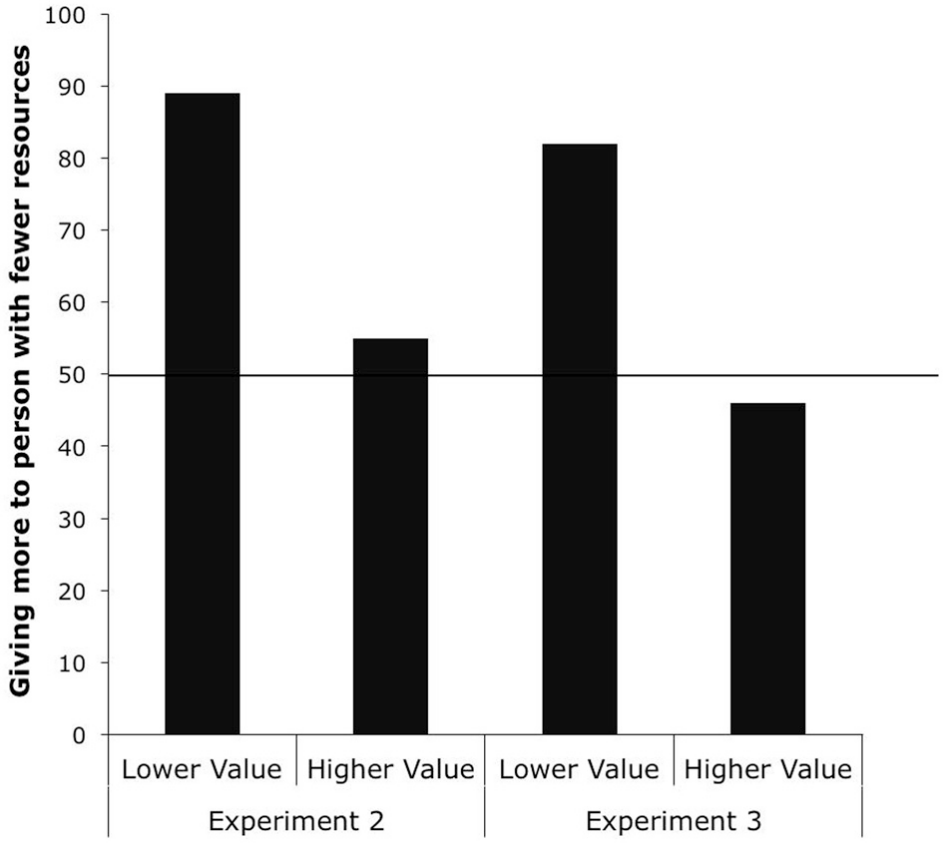
**Percentage of children giving two resources to the recipient with fewer resources in the Lower and Higher Value Conditions from Experiments 2 and 3**.

Again, because very few children chose the option of giving two resources to the person with more resources (only one child who was in the Higher Value Condition and no children in the Lower Value Condition), we again conducted our analyses focusing on the strategies that children used—sharing equally themselves by giving one to each recipient, or giving two to the person with fewer resources^1^. A 2 × 2 Yates-corrected χ^2^ test revealed that children in the Higher Value Condition were more likely to give one resource to each child than children in the Lower Value Condition, χ^2^ (1, *N* = 55) = 6.28, *p* = 0.013, see Figure 2. When the resources children shared were more valuable than the resources shared unequally by the experimenter, children shifted their preference from giving two to the person with fewer resources to giving one resource to each recipient.

We next conducted binomial tests to compare children's responses to chance responding. A binomial test on children's choices in the Higher Value Condition revealed that children did not show a preference, with about half the children giving two to the person with fewer resources (15 out of 27) and about half of the children giving one to each recipient (12 out of 27), *p* = 0.701. However, the binomial test on the Lower Value Condition revealed that children chose to give two resources to the recipient with fewer resources (25 out of 28) more often than giving one to each recipient (3 out of 28), *p* < 0.001.

### Discussion

We again found that children correct previous inequalities in order to minimize inequalities in outcomes between recipients, and do so by using the value of the resources at their disposal. When children were presented with an inequality involving high value resources, but only had a few low value resources with which to address it, children gave two to the person with fewer resources since this was the best way to minimize inequality. However, when children were presented with an inequality involving low value resources, but only had a few high value resources with which to address it, children were much less likely to give two to the person with fewer resources. Importantly, in both conditions children were dividing resources that were different from the resources that were distributed by the experimenter, so the results cannot be explained by confusion involving the distribution of different resources (which was common to both conditions). In fact, the same resources were used in both conditions; what differed between conditions was which resource was distributed by the experimenter and which was distributed by the participant. These results suggest that children focus on trying to equalize outcomes, and that they do so by using the value of resources.

Although the results thus far are consistent with children using value to determine how to minimize inequality in outcomes, children could be using an even simpler heuristic—size of resource. In Experiments 1 and 2, the high value resource was physically larger than the low value resource, and so children may have been using resource size, not value, to guide their decisions. In Experiment 3, we dealt with this confound by matching the volume and surface area of the high and low value resources.

## Experiment 3

### Methods

#### Participants

Participants included 56 children aged 6 to 8 years old. Of these participants, 28 were in the Lower Value Condition (*M* = 7 years, 5.5 months, *SD* = 12 months; 13 females) and 28 were in the Higher Value Condition (*M* = 7 years, 3.5 months, *SD* = 11 months; 12 females).

#### Procedure

The procedure for Experiment 3 was the same as Experiment 2, except that we used different resources: chocolate bars, and pieces of cardboard cut to the same size as the chocolate bars. In the Lower Value Condition, the experimenter gave out high value resources (four chocolate bars; three to one recipient and one to the other) and the participant gave out two lower value rewards (two pieces of cardboard). In the Higher Value Condition, the experimenter gave out lower value resources (four pieces of cardboard; three to one recipient and one to the other) and the participant gave out two higher value rewards (two chocolate bars). If children in the previous experiments were trying to equate the volume or surface area of the resources, then children should behave similarly in the Higher and Lower Value Conditions here. However, if children in the previous experiments were trying to minimize inequality in outcomes by using value, then they should be more likely to share equally themselves by giving one resource to each recipient when distributing the higher value reward as opposed to the lower value reward (for empirical verification that children see the chocolate bars as more valuable than cardboard, see Experiment 4).

### Results

Again, because no children chose the option of giving more resources to the recipient with more resources, we conducted our analyses on children's two strategies of giving more to the person with fewer resources and giving one to each recipient. A Yates-corrected χ^2^ test revealed that children in the Higher Value Condition were more likely to give one resource to each recipient than children in the Lower Value Condition, χ^2^ (1, *N* = 56) = 6.3, *p* = 0.012. As the value of the resource increased, children shifted their preference from giving two to the recipient with fewer resources to giving one resource to each recipient (see Figure 2).

We next conducted binomial tests to compare children's responses to chance responding. A binomial test on children's choices in the Higher Value Condition revealed that children did not show a preference, with about half the children giving two to the recipient with fewer resources (13 out of 28) and about half of the children giving one to each recipient (15 out of 27), *p* = 0.850. However, a binomial test on the Lower Value Condition revealed that children chose to give two to the recipient with fewer resources (23 out of 28) more often than giving one to each recipient (5 out of 28), *p* < 0.001.

### Discussion

These results again indicate that children are motivated to create equal outcomes, not just to give equally themselves, and that they use value, not just the volume or surface area of resources, to decide how to create equal outcomes for others. When a resource was of lower value than the resources involved in the original inequality, children gave more to the recipient who received fewer resources originally in order to correct the previous inequality. However, children were much less likely to give more to the recipient with fewer resources if the resources they were distributing were more valuable than those involved in original inequality, presumably because they understand that this would make things more unequal.

However, one limitation of Experiments 1 through 3 is that we did not have an empirical measurement of value. We deliberately chose resources that seemed more valuable to adults; however, we do not know if children actually think these resources are more valuable. In Experiment 4 we ask children explicitly about which items they think that another child would prefer and how many of the less preferred items they think one would need to trade in order to get the more preferred item.

One other open question from the previous experiments is whether children distributed high value resources differently then low value resources because they treat high value resources differently in general or because they noticed that the high value resources were more valuable than the originally distributed resources. Perhaps children just maintain the status quo by sharing equally when they are given certain resources to share, regardless of the value of resources shared by the experimenter. To examine this possibility in Experiment 4 we had children distribute the high value resource from Experiment 3 (a chocolate bar) in a situation in which equal sharing was not the option that minimized inequality—where an experimenter shared three chocolate bars with one recipient and one chocolate bar with the other. If children treat certain resources differently regardless of context, then they should give one chocolate bar to each recipient as they did in Experiment 2 and 3 when sharing chocolate bars. However, if what matters is the relationship between the value of resources already distributed and the resource children are sharing, then they should now give two chocolate bars to the person with fewer resources because this would minimize inequality between the two recipients.

## Experiment 4

### Methods

#### Participants

Participants included 28 children aged 6 to 8 years old (*M* = 7 years, 4 months, *SD* = 11 months; 13 females).

#### Procedure

The procedure for Experiment 4 was the same as Experiment 1 Equal Value Condition, except that the equal value resource was now the chocolate bars from Experiments 2 to 3 rather than erasers. That is, the experimenter gave out four chocolate bars, three to one recipient and one to the other, and the participant gave out two of the same kind of chocolate bar. In the previous experiments chocolate bars were treated as a high value resource in comparison to small fruit candies and cardboard. Therefore, if children are simply more inclined to maintain the status quo when distributing objectively valuable resources like chocolate bars, then we should see children giving one chocolate bar to each recipient as they had in Experiments 2 and 3. However, if what children are attempting to do is to equate value, then we should see them giving two candy bars to the recipient with fewer candy bars.

After completing the Equal Value Condition, children completed an explicit measure of value. We asked children to decide which resource they thought Mark would prefer, resource *X* or resource *Y*—which corresponded to the pairs of resources used in Experiments 1 through 3. Children were asked about the four resource pairs in the following order: eraser vs. Play-Doh, eraser vs. $20 bill, chocolate bar vs. small fruit candy, chocolate bar vs. piece of cardboard (or the reverse order, counterbalanced between participants). The items were placed in front of children and children were asked, “Which do you think Mark would rather have?” Children indicated their choice by pointing at one of the two resources. After answering which one Mark would prefer, children were asked how many of the resource they did not choose (the one they thought Mark would not prefer) Mark would need to trade in order to get one of the chosen resource. The trading measure was designed to produce a rough estimate of how much more valuable children thought one resource was.

### Results and discussion

Again, because no children chose the option of giving more resources to the recipient with more resources, we conducted our analyses on children's two strategies of giving more to the recipient with fewer resources and giving one to each recipient. We conducted binomial tests to compare children's responses to chance responding. A binomial test on children's choices in the equal value condition revealed that that children chose to give two to the recipient with fewer resources (24 out of 28) more often than giving one to each recipient (4 out of 28), *p* < 0.001. This result indicates that children do not simply maintain the status quo when distributing chocolate bars, a high value resource from Experiment 2 and 3. Children are perfectly willing to share unequally by giving more to the person with fewer resources, disrupting the status quo, when the chocolate bars are the same value as the resource shared unequally by the experimenter.

We next conducted binomial tests on children's responses to which resource Mark would prefer. Children thought that Mark would prefer: a jar of Play-Doh to an eraser (26 out of 28), *p* < 0.001, a $20 bill to an eraser (28 out of 28), *p* < 0.001; a chocolate bar to a piece of cardboard (27 out of 28), *p* < 0.001; and a chocolate bar to a small fruit candy (24 out of 28), *p* < 0.001. These results indicate that our intuitions about children's valuation of these objects was correct—children thought that the items we labeled as higher value resources in Experiments 1 through 3 were in fact more preferred than the items we labeled as lower value resources. We next examined how many low value resources children thought one would need to trade to get one of the high value resources. We found that children thought that one would need to trade, on average, 8.5 erasers to get one jar of Play-Doh, 17 erasers to get one $20 bill, 10 small fruit candies to get one chocolate bar, and 26.5 pieces of cardboard to get one chocolate bar. It is worth noting that in order to reduce skew on the cardboard/chocolate item we had to code five of the children's responses as “100” because they either gave very large numbers (two children said one thousand and one child said one billion) or they stated that no amount of the cardboard could be traded for a chocolate bar (*N* = 2).

## General discussion

These experiments demonstrated that 6- to 8-year-olds are more concerned with making the outcome of a resource distribution equal than with giving equally themselves. They also demonstrated that children consider value when responding to inequalities. Experiment 1 showed that children will give unequally themselves in order to minimize inequality of outcome. Children gave two resources to the recipient with fewer resources so that both recipients would have three resources, rather than giving equally themselves and maintaining the inequality. This result is consistent with past research demonstrating that children will give unequally in some circumstances, such as when others have done more work (Damon, 1977; Sigelman and Waitzman, 1991); however, these results are the first direct demonstration that children will correct unequal distributions by sharing unequally with others.

Experiments 1 and 2 also investigated what measure children use to determine how best to minimize inequality. These experiments illustrated that children use the *value* of resources, not just the count, to minimize inequality between others. They did not opt to give one person two high value resources (equalizing the count of resources) to correct past unequal sharing of a low value resource, and instead were more likely to give one high value resource to each recipient. Experiment 3 further confirmed that children were using value, not resource size, as a guide for how to share resources with others. In Experiment 4 children were asked to make explicit judgments about which resources they thought another child would prefer. These explicit judgments provided an empirical confirmation that our high value resources were actually valued more highly than resources that we labeled as lower value resources.

It is worth noting that while children became less likely to give both resources to the recipient with fewer resources as the value of the new resources increased, in Experiments 2 and 3 about half the participants still attempted to equate resource count rather than resource value when sharing the high value resource (large chocolate bar). It is unclear why children gave mixed responses in this case, though there are several possibilities. One possibility is that some children placed different value on the items they were asked to share. If children thought the chocolate bars were about as valuable as the fruit candies or cardboard, then it would be unsurprising that they attempted to equate count rather than value. However, a more likely possibility is that children did not know which norm to apply to this situation and so were forced to choose between two conflicting norms: should I equalize the count or value of the resources? This conflict in norms may have made children confused about what to do and led to their chance responding when distributing the higher value rewards. However, what is important about these results is that children did differentiate between distributing resources that had higher and lower value than the original inequality, suggesting that at least some children take resource value into account when deciding how to minimize inequality in outcomes between others.

The current findings are interesting to consider in light of recent work demonstrating that children are fair partly in order to signal to others that they are fair. Shaw et al. (in press) found that 6- to 8-year-old children were very fair when the other option was to appear unfair to an experimenter (see also Blake and McAuliffe, 2011), but were considerably less fair when they did not risk appearing unfair. The paradigm developed here could be used to investigate if children will use ambiguous norms, like those investigated in the current studies, to their advantage in order to appear fair while getting more for themselves. For example, imagine we repeated the High Value Condition from Experiment 1, but the participant was the recipient who received more erasers (low value rewards). We could then ask the participant to distribute two $20 bills (high value rewards) between him- or herself and another recipient. Here, it seems likely that children would do the fair thing and give one $20 bill to themselves and one to the other recipient because they would have no way to justify giving two $20 bills to themselves. Next, imagine we repeated the High Value Condition from Experiment 1, but that this time the participant received fewer erasers than the other recipient and was again asked to distribute two $20 bills. In this case, children might be more likely to take the two $20 bills for themselves since they would have the plausible justification that they were simply trying to equate the count of resources. These results would demonstrate that children can use different norms and plausible deniability to justify their own selfishness, just as adults do (Dana et al., 2007; Andreoni and Bernheim, 2009). Future research should investigate this possibility.

The results of our experiments demonstrate that the value of resources influences children's sharing behavior, but they do not address how children determine the value of resources in the first place. The first strategy that children likely use to determine value is to simply use their own preferences as a guide for how to share with others. That is, they know what they like, and think that the things they like are valuable and that the things they dislike are not valuable. This strategy is likely a large part of children's early understanding of value, but as they get older they may use more sophisticated variables to determine how resources are valued. One possibility is that children use some aggregate sense of others' preference for a resource, analogous to the adult concept of demand—recognizing that the more others want a resource, the more valuable it is (Baumol, 1972). A second possibility is that children use resource scarcity to determine value, recognizing, as adults do, that rare things are more valuable than things that are commonly available (Lynn, 1991). Yet another possibility is that children use effort expended to obtain a resource to determine resource value; all else being equal, they may assume that if a person worked harder to make or obtain a resource, that resource is more valuable. It is likely that children, like adults (Baumol, 1972), use some combination of these factors to determine a resource's value, and as they get older they incorporate more of these sophisticated principles to determine resource value.

Now that we know that children can use value to guide their equality judgments, we can investigate whether or not children use value in other domains such as trade. Trade is ubiquitous in modern society and simpler forms of bartering were also very prevalent before the advent of currency (Fagan, 1969; Hill and Kaplan, 1993). Being able to equate resources that differ in value is essential for participating in trade, both modern forms of trade between nations and simpler forms of bartering (Krugman, 1979; Hill and Kaplan, 1993). Without some sense of value it would be impossible to determine when one should and should not trade with another person. Anecdotally, children trade a number of resources, from baseball cards to lunchtime snacks to Silly Bands—children seem well acquainted with trade. Yet it is unclear whether these trades are simply based on personal preference or on some understanding of resource value. How do children reconcile others' personal preferences with the objective value of resources? Is subjective or objective value given more weight? Can children capture gains from trade (Krugman, 1979)? Understanding children's early notions of trade may provide some insight into how they grow into adults who perform more sophisticated exchanges.

Despite remaining questions, the current research demonstrates that children do not treat all inequalities equally—they use resource value, rather than just resource count, when deciding how to share with others.

### Conflict of interest statement

The authors declare that the research was conducted in the absence of any commercial or financial relationships that could be construed as a potential conflict of interest.

## Footnotes

^1^We analyze the results here without including the one child who gave the non-standard response of giving more to the person with more resources, but the pattern of results remain the same if we conservatively run the analyses counting this child as having given more to the person with fewer resources.

## References

[B1] Andreoni J. Bernheim D. B. (2009). Social image and the 50–50 norm: a theoretical and experimental analysis of audience effects. Econometrica 77, 1607–1636 10.3982/ECTA7384

[B2] Baumard N. Mascaro O. Chevallier C. (2012). Preschoolers are able to take merit into account when distributing goods. Dev. Psychol. 48, 492–498 10.1037/a0026598 22148948

[B3] Baumol W. J. (1972). Economic Theory and Operations Analysis. Englewood Cliffs, NJ: Prentice-Hall.

[B4] Birch L. Billman J. (1986). Preschool children's food sharing with friends and acquaintances. Child Dev. 57, 387–395 10.2307/1130594

[B5] Blake P. R. McAuliffe K. (2011). “I had so much it didn't seem fair”: eight-year olds reject two forms of inequity. Cognition 120, 215–224 10.1016/j.cognition.2011.04.006 21616483

[B6] Blake P. Rand D. (2010). Currency value moderates equity preference among young children. Evol. Hum. Behav. 31, 210–218 10.1016/j.evolhumbehav.2009.06.01221616483

[B7] Boyd R. Richerson P. J. (2005). The Origin and Evolution of Cultures. New York, NY: Oxford University Press.

[B8] Brown T. C. (1984). The concept of value in resource allocation. Land Econ. 60, 231–246 10.2307/3146184

[B9] Burgoyne C. B. Lea S. E. G. (2006). Money is material. Science 314, 1091–1092 10.1126/science.1135429 17110558

[B10] Cook K. S. Hegtvedt K. A. (1983). Distributive justice, equity, and equality. Ann. Rev. Soc. 9, 217–241 10.1146/annurev.so.09.080183.001245

[B12] Damon W. (1977). The Social World of the Child. San Francisco: Jossey-Bass.

[B13] Dana J. Weber R. Kuang J. (2007). Exploiting moral wiggle room: experiments demonstrating an illusory preference for fairness. Econ. Theory 33, 67–80 10.1007/s00199-006-0153-z

[B14] Dawes C. Fowler J. Johnson T. McElreath R. Smirnov O. (2007). Egalitarian motives in humans. Nature 446, 794–796 10.1038/nature05651 17429399

[B15] DeVoe S. E. Iyengar S. S. (2010). Medium of exchange matters what's fair for goods is unfair for money. Psychol. Sci. 21, 159–162 10.1177/0956797609357749 20424037

[B16] Fagan B. M. (1969). Early trade in south central Africa. Archaeology 2, 44–50

[B18] Fehr E. Schmidt K. M. (1999). A theory of fairness, competition, and cooperation. Q. J. Econ. 114, 817–868 10.1162/003355399556151

[B19] Fiske A. P. (1992). The four elementary forms of sociality: framework for a unified theory of social relations. Psychol. Rev. 99, 689–723 10.1037/0033-295X.99.4.689 1454904

[B20] Geraci A. Surian L. (2011). The developmental roots of fairness: infants' reactions to equal and unequal distributions of resources. Dev. Sci. 14, 1012–1020 10.1111/j.1467-7687.2011.01048.x 21884317

[B21] Gurven M. (2004). To give or to give not: an evolutionary ecology of human food transfers. Behav. Brain Sci. 27, 543–583 10.1017/S0140525X04000123

[B22] Gurven M. (2006). The evolution of contingent cooperation. Curr. Anthropol. 47, 185–192 10.1086/499552

[B23] Harbaugh W. T. Krause K. Berry T. R. (2001). GARP for kids: on the development of rational choice behavior. Am. Econ. Rev. 91, 1539–1545 10.1257/aer.91.5.1539

[B24] Hill K. Kaplan H. (1993). On why male foragers hunt and share food. Curr. Anthropol. 34, 701–710 10.1086/204213

[B25] Hook J. Cook T. D. (1979). Equity theory and the cognitive ability of children. Psychol. Bull. 86, 429–445 10.1037/0033-2909.86.3.42922858091

[B26] Kenward B. Dahl M. (2011). Preschoolers distribute resources according to recipients' moral status. Dev. Psychol. 47, 1054–1064 10.1037/a0023869 21604863

[B27] Krugman P. (1979). Increasing returns, monopolistic competition, and international trade. J. Int. Econ. 9, 469–479 10.1016/0022-1996(79)90017-5

[B28] Lerner M. (1974). The justice motive: “Equity” and “parity” among children. J. Pers. Soc. Psychol. 29, 539–550 10.1037/h0036206

[B29] Libby W. Garrett J. (1974). Role of intentionality in mediating children's responses to inequity. Dev. Psychol. 10, 294–297 10.1037/h0035985

[B30] Lynn M. (1991). Scarcity effect on value: a quantitative review of the commodity theory literature. Psychol. Market. 8, 43–57 10.1002/mar.4220080105

[B31] Olson K. R. Spelke E. S. (2008). Foundations of cooperation in young children. Cognition 108, 222–231 10.1016/j.cognition.2007.12.003 18226808 PMC2481508

[B32] Mills J. Clark M. S. (1994). Communal and exchange relationships: controversies and research, in Theoretical frameworks for personal relationships, eds Erber R. Gilmour R. (Hillsdale, NJ: Erlbaum), 29–42

[B33] Moore C. (2009). Fairness in children's resource allocation depends on the recipient. Psychol. Sci. 8, 944–948 10.1111/j.1467-9280.2009.02378.x 19515118

[B34] Novakova J. Flegr J. (2013). How much is our fairness worth? The effect of raising stakes on offers by proposers and minimum acceptable offers in dictator and ultimatum games. PLoS ONE 8:e60966 10.1371/journal.pone.0060966 23580080 PMC3620327

[B36] Schmidt M. F. Sommerville J. A. (2011). Fairness expections and altruistic sharing in 15-month-old human infants. PLoS ONE 6:e23223 10.1371/journal.pone.0023223 22003380 PMC3188955

[B37] Shaw A. DeScioli P. Olson K. R. (2012). Fairness versus favoritism in children. Evol. Hum. Behav. 33, 736–745 10.1016/j.evolhumbehav.2012.06.001

[B38] Shaw A. Knobe J. (2013). Not all mutualism is fair, and not all fairness is mutualistic. Behav. Brain Sci. 36, 100–101 10.1017/S0140525X12000878 23445600

[B39] Shaw A. Montinari N. Piovesan M. Olson K. R. Gino F. Norton M. I. (in press). Children develop a veil of fairness. J. Exp. Psychol. Gen. [Epub ahead of print]. 10.1037/a0031247 23317084

[B40] Shaw A. Olson K. R. (2012). Children discard a resource to avoid inequity. J. Exp. Psychol. Gen. 141, 382–395 10.1037/a0025907 22004168

[B41] Sigelman C. K. Waitzman K. A. (1991). The development of distributive justice orientations: contextual influences on children's resource allocations. Child Dev. 62, 1367–1378 10.2307/1130812 1786721

[B42] Sloane S. Baillargeon R. Premack D. (2012). Do infants have a sense of fairness? Psychol. Sci. 23, 196–204 10.1177/0956797611422072 22258431 PMC3357325

[B43] Sommerville J. A. Schmidt M. F. Yun J. E. Burns M. (2012). The development of fairness expectations and prosocial behavior in the second year of life. Infancy 18, 40–66 10.1111/j.1532-7078.2012.00129.x

[B45] Walster E. Walster G. W. Berscheid E. (1978). Equity Theory and Research. Boston: Allyn & Bacon

[B46] Xiao E. Bicchieri C. (2010). When equality trumps reciprocity. J. Econ. Psychol. 31, 456–470

